# Evaluating Somatic Mutational Contamination in Large-Scale Germline Genomic Studies

**DOI:** 10.3390/biology15141204

**Published:** 2026-07-21

**Authors:** Xiangwen Ji, Xueke Bai, Guangda He, Kai Yan, Edwin Wang, Yi-Da Tang, Liang Chen, Qinghua Cui

**Affiliations:** 1 Department of Cardiology and Institute of Vascular Medicine, State Key Laboratory of Vascular Homeostasis and Remodeling, Peking University Third Hospital, 49 Huayuanbei Road, Beijing 100191, China; jxw01@pku.edu.cn (X.J.); tangyida@bjmu.edu.cn (Y.-D.T.); 2 National Clinical Research Center for Cardiovascular Diseases, State Key Laboratory of Cardiovascular Disease, Fuwai Hospital, National Center for Cardiovascular Diseases, Chinese Academy of Medical Sciences and Peking Union Medical College, Beijing 100037, China; baixueke@fuwai.com (X.B.); heguangda@fuwai.com (G.H.); 3 Department of Biochemistry and Molecular Biology, Medical Genetics, and Oncology, Cumming School of Medicine, University of Calgary, Calgary, AB T2N 1N4, Canada; yankaimutd@163.com (K.Y.); edwin.wang@ucalgary.ca (E.W.); 4 School of Sports Medicine, Wuhan Sports University, No. 461 Luoyu Rd. Hongshan District, Wuhan 430079, China; 5 Department of Biomedical Informatics, State Key Laboratory of Vascular Homeostasis and Remodeling, School of Basic Medical Sciences, Peking University, 38 Xueyuan Rd, Beijing 100191, China

**Keywords:** genomics, germline mutation, somatic mutation, mutational signature, UK Biobank, smoking

## Abstract

Large-scale genomic initiatives like the UK Biobank have revolutionized our understanding of human disease. These studies typically assume that blood-derived DNA faithfully reflects an individual’s germline genome. However, this assumption is challenged by somatic mutations arising from processes like clonal hematopoiesis. Although standard bioinformatics pipelines employ variant allele frequency (VAF)-based filtering to mitigate such contamination, the efficacy of these approaches requires systematic evaluation. By systematically analyzing germline genome data from large cohorts through applications of mutational signatures, we revealed critical limitations in current filtering methodologies. We found that the mutational spectrum of rare “germline” variants is highly similar to that of somatic mutations. Furthermore, we uncovered that these variants show significant associations with phenotypes such as age, sex, and smoking status, established drivers of somatic mutagenesis. Notably, our multivariable regression models estimated that these somatic artifacts contribute to a substantial excess burden, such as 4.73 mutations per megabase (mut/Mb) in males compared to females, a magnitude exceeding the mutation burden of many cancers. Although the precise absolute size of this contamination may vary depending on specific pathologies and individual environmental exposures, this persistent somatic contamination introduces substantial confounder effects, posing a risk of spurious associations and reverse causality in genetic studies. Our work underscores the urgent reconsideration of two fundamental aspects of genomic research: (1) refinement of variant filtering strategies to better distinguish true germline variants from somatic contaminants, and (2) incorporation of somatic mutagenesis factors as essential covariates in study design. Our findings provide basic guidance for improving the accuracy and interpretability of large-scale genomic studies.

## Simple Summary

Large-scale genomic studies often rely on blood samples to determine a person’s inherited DNA mutations. However, blood cells accumulate non-inherited somatic mutations over a lifetime due to aging and environmental exposures like smoking. These acquired changes contaminate the data, which may lead to incorrect conclusions about inherited health risks. This study aimed to systematically evaluate the extent of this contamination and its impact on genomic research. By analyzing large global genomic databases, we discovered that current filtering methods may not fully separate true inherited mutations from somatic mutations. The “inherited” mutations showed associations with age, sex, and smoking history that are suggestive of somatic confounding. Specifically, we found that genome-wide association studies of smoking and smoking-related diseases may be susceptible to biased associations due to this contamination. Our findings conclude that somatic mutations distort the accuracy of large-scale genomic studies, highlighting the urgent need to develop better filtering technologies and to account for lifestyle factors in genetic study designs.

## 1. Introduction

The application of whole-exome sequencing (WES) and whole-genome sequencing (WGS) to large-scale population cohorts, such as the UK Biobank (UKB), has significantly advanced our understanding of the genetic basis of complex diseases [1,2,3,4]. These massive datasets form the cornerstone of exome-wide association studies (ExWASs), genome-wide association studies (GWASs), and protein quantitative trait loci (pQTLs) studies, which aim to identify genetic loci associated with specific traits or diseases by analyzing genetic variation among individuals [5,6,7]. A central premise of these studies is that blood-derived DNA accurately represents an individual’s germline (i.e., constitutional) genome.

However, this foundational assumption is challenged by the accumulation of somatic mutations in normal tissues throughout an individual’s lifespan [8,9]. Unlike germline variants, which are inherited and present in all cells, somatic mutations are acquired post fertilization and remain restricted to specific somatic lineages or localized clones [10]. These mutations consist predominantly of single-base substitutions (SBSs), along with occasional insertions, deletions, other types of base substitutions (e.g., double-base substitutions), and structural variants [11,12]. The etiologies of these mutations are fundamentally shaped by a combination of endogenous factors, such as spontaneous DNA replication errors and DNA damage repair errors, as well as exogenous environmental exposures, including tobacco smoke and other mutagens [11]. In the hematopoietic system, healthy hematopoietic stem cells acquire approximately 20 somatic mutations per year [13,14]. In clonal hematopoiesis (CH), hematopoietic stem cells acquire somatic mutations that confer a selective advantage, leading to their clonal expansion [15]. Consequently, when whole blood is used as the DNA source in WES or WGS studies, these somatic mutations will be admixed into the DNA samples for sequencing. This admixture then introduces a form of “biological noise”. Specifically, because these somatic mutations, often referred to as CH of indeterminate potential (CHIP), are strongly associated with age, sex, and environmental exposures like smoking [16], they become disproportionately enriched in populations with these risk factors. Consequently, in GWASs evaluating diseases sharing these same risk factors (e.g., smoking addiction or lung cancer), these misclassified somatic variants will exhibit unequal allele distributions between cases and controls. This systematic bias allows CHIP variants to masquerade as inherited risk alleles and create spurious genetic associations [17].

To address this challenge, researchers commonly employ filtering strategies based on variant allele frequency (VAF) to distinguish between germline and somatic variants. The rationale behind this approach is that a true heterozygous germline variant is expected to have a VAF of approximately 50%, whereas a homozygous germline variant will have a VAF of 100%. In contrast, the VAF of a CHIP mutation is typically substantially lower than 50% [18]. Therefore, bioinformatics pipelines [7,19] typically set a VAF threshold (e.g., ≥20%) and/or utilize a binomial test to remove variants with a VAF significantly below 50%, thereby enriching for high-confidence germline variants. Additionally, metrics such as sequencing depth are considered standard practice to effectively exclude somatic interference and purify the germline signal [7,20].

Although current variant filtering methods are intuitively designed and widely adopted, our study reveals their critical limitation: they fail to fully purge somatic mutational contamination from germline datasets. Here, we introduce a novel perspective by systematically evaluating the frequency-dependent spectral characteristics of WES and WGS data from large cohorts, including the UKB, The Cancer Genome Atlas (TCGA), and the 1000 Genomes Project, we identified persistent somatic mutational signatures classified as germline, particularly within rare variant subsets. Strikingly, these ostensibly germline variants exhibit molecular profiles characteristic of somatic mutagenesis, suggesting substantial admixture between true germline variants and somatic mosaic events. Such misclassification poses a serious analytical challenge, as residual somatic variants masquerading as germline variants could introduce spurious associations in genetic studies, which may ultimately confound our interpretation of disease etiology. Our findings underscore the imperative to rigorously quantify and account for somatic contamination in germline genomic datasets. Addressing this underappreciated source of bias is essential for enhancing the precision and reproducibility of large-scale genomic studies.

## 2. Materials and Methods

### 2.1. Collection of Germline and Somatic Mutation Data

The UKB stands as a large-scale, prospective cohort study and a comprehensive global biomedical resource. It houses an extensive collection of genetic, proteomic, and phenotypic data from its participants. Between 2006 and 2010, a total of 502,156 individuals, aged 40–69 at baseline, were enrolled from 22 different centers across the UK. For the UKB WES cohort, exomes were captured using the IDT xGen Exome Research Panel v1.0 (Integrated DNA Technologies, Coralville, IA, USA) with supplemental probes and sequenced with 75 bp paired-end reads on the Illumina NovaSeq 6000 (Illumina, San Diego, CA, USA) platform [1]. The sequencing data were processed through the Original Quality Functional Equivalent (OQFE) pipeline, and germline variants were subsequently called using DeepVariant [21] (v0.10.0, reference genome: GRCh38). Finally, variant information was available for 469,881 participants. Access to UK Biobank data can be requested through the standard application process at https://www.ukbiobank.ac.uk/use-our-data/apply-for-access/ (accessed on 20 April 2023). This study utilized data from the UKB resource under approved application number 87841.

In addition to the UKB WES cohort, we applied for access to the germline mutation data of 10,389 TCGA patients from a previous study [22] (reference genome: GRCh37). Germline variant data from the 1000 Genomes Project [23] Phase 1 (release: 20110521, reference genome: GRCh37) and Phase 3 (release: 20130502, reference genome: GRCh37), based on whole-genome sequencing (WGS), were downloaded from the official FTP site at https://ftp.1000genomes.ebi.ac.uk/vol1/ftp/release/ (accessed on 22 November 2022).

The Catalogue of Somatic Mutations in Cancer (COSMIC) is the most comprehensive and detailed resource for somatic mutations in human cancer [24]. We downloaded frequency data for coding mutations in hematopoietic and lymphoid tissues from COSMIC v99 (reference genome: GRCh38) at https://cancer.sanger.ac.uk/cosmic (accessed on 19 January 2024).

### 2.2. Germline Variant Filtering

To filter germline variants, we adopted a strategy similar to that used in our previous work [20]. First, a minimum sequencing depth of 20 was required to ensure sequencing reliability. The VAF was calculated as the ratio of variant allele depth (AD) to total depth (DP), i.e., VAF = AD/DP. To exclude potential somatic mutations, such as those associated with CHIP, which typically present with low VAF, we fitted the VAF distribution of all variants using a Gaussian Mixture Model (GMM). In our previous study, we defined variants with a VAF between 0.422 and 0.54 or ≥0.9 as high-confidence germline mutations.

Furthermore, to assess the robustness of our results, we also tested five alternative filtering strategies: (1) DP ≥ 10, and either 0.422 ≤ VAF ≤ 0.54 or VAF ≥ 0.9; (2) DP ≥ 20, VAF ≥ 0.2, and binomial test *p*-value ≥ 1 × 10^−6^ for heterozygous; (3) DP ≥ 10, VAF ≥ 0.2, and binomial test *p*-value ≥ 1 × 10^−6^ for heterozygous; (4) DP ≥ 20 and AD ≥ 5; (5) DP ≥ 10 and AD ≥ 3. Among these, using a binomial test to retain variants whose allele frequencies are not strongly significantly (*p* ≥ 1 × 10^−6^) different from a true heterozygous state (VAF = 50%) is a common strategy in UKB-based studies to exclude somatic mosaicism [7,19]. Based on the filtering results, our primary strategy proved to be the most stringent, yielding the lowest number of distinct variants (1,447,265) and total variant calls (9,571,462,124).

As AD and DP information was not available for the 1000 Genomes Project data, we could not apply the aforementioned filters. However, these data have undergone rigorous quality control by the 1000 Genomes Project Consortium [23], lending confidence to the reliability of the variant calls. Given that these cohorts are only used for the qualitative evaluation of mutation spectra trends, rather than for quantitative burden regression, this difference is deemed acceptable.

To define rare variants, we initially selected an allele frequency (AF) threshold of ≤0.1%, a widely accepted standard in population genetics for identifying rare variation. To ensure our findings were not artifacts of this specific threshold, we systematically evaluated the stability of our main conclusions across a range of AF cutoffs (from ≤10% down to ≤0.01%).

### 2.3. Correlation Between Mutation Frequency and Substitution Type

For each distinct mutation, the mutation frequency was calculated as the percentage of individuals carrying the variant relative to the total number of individuals in the respective database.

(1) $$\mathrm{mutation}\;\mathrm{frequency}=\frac{number\;of\;individuals\;with\;the\;mutation}{number\;of\;individuals\;in\;the\;database}$$

To assess frequency-dependent trends, all distinct mutations were grouped into contiguous frequency bins along a logarithmic scale. To ensure statistical robustness and verify that the observed trends were not artifacts of a specific interval choice, this binning procedure was systematically repeated using varying logarithmic interval sizes (bin sizes of 10, 10^1/2^, 10^1/3^, 10^1/4^, 10^1/5^). For example, if a bin size of 10^1/2^ were used, the mutations would be grouped into these bins according to their mutation frequency: (10 × 10^1/2^%, 100%], (10%, 10 × 10^1/2^%], (10^1/2^%, 10%], (1%, 10^1/2^%], (0.1 × 10^1/2^%, 1%], (0.1%, 0.1 × 10^1/2^%], etc.

Within each frequency bin, we calculated the percentages of each of the six base substitution types (C>A, C>G, C>T, T>A, T>C, and T>G). This was defined as the number of distinct variants of a specific substitution type divided by the total number of distinct variants falling into that specific frequency bin. This calculation method was similarly applied to specific trinucleotide contexts (e.g., the percentage of A[C>T]G context among all mutations within a bin). We performed Spearman’s rank correlation analysis assessing the relationship between the ordinal rank of the logarithmic frequency bins and the corresponding percentage of the mutation type within those bins.

### 2.4. Germline Genome Pattern (GGP) Analysis

Our GGP analysis followed a procedure similar to SigProfiler [25] (v1.2.1) and our previous study [20]. Specifically, for each SBS, we identified the base substitution and its immediate 5′ and 3′ flanking nucleotides. This defined a trinucleotide context, resulting in 96 possible mutation types for pyrimidine (C/T) substitutions (4 types of 5′ base × 6 types of substitution × 4 types of 3′ base). Substitutions involving purines (G/A) were reverse complemented to be represented by their pyrimidine-based equivalents.

For each sample, all SBS germline variants were classified into one of the 96 trinucleotide contexts, and the counts for each context were tallied. For a cohort of N samples, this process generates a 96 × N matrix, denoted as X. Using a Non-negative Matrix Factorization (NMF) algorithm [26], matrix X can be decomposed into two matrices: a 96 × k signature matrix W and a k × N activity matrix H, such that

(2) X ≈ W · H.

The matrices can be scaled such that each column of W sums to 1, allowing it to be interpreted as a probability distribution representing a distinct mutational signature. Consequently, each column in matrix H contains k values, representing the activities of the k signatures (e.g., GGP A, GGP B, etc.) in the corresponding sample. For new data, the signature activities can be determined by fitting a new activity matrix H while keeping the signature matrix W fixed.

To determine the optimal number of signatures, k, we iterated over k from 1 to 25. For each k, we performed a resampling procedure 100 times. In each resampling iteration, a new mutation count matrix was generated by sampling from multinomial distributions based on each sample’s original mutation profile. NMF was applied to each resampled matrix to extract 100 sets of k signatures. These 100 × k signatures were then clustered into k groups using K-means clustering. The Hungarian algorithm [27] was used to match signatures across iterations, minimizing cosine distances to cluster centroids. The mean silhouette coefficient and minimum silhouette coefficient (using cosine distance) were used to evaluate clustering quality and stability. For k = 1, both coefficients were set as 1. We selected the largest k for which the mean and minimum coefficients remained above 0.8 and 0.2, respectively. The final k unsupervised extracted signatures were defined by averaging the 100 signatures within each cluster. These computations were performed using Python (v3.13). NMF was implemented with torchnmf (v0.3.5), K-means clustering and silhouette coefficient were implemented with scikit-learn [28] (v1.7.0), the Hungarian algorithm was implemented with SciPy [29] (v1.15.3), and multinomial sampling was implemented with NumPy [30] (v2.3.0).

After obtaining the unsupervised signatures, we decomposed them into known COSMIC mutational signatures (v3.4) using SigProfilerAssignment [31] (v0.2.3). A non-negative least squares (NNLS) approach, with a regularization penalty to prevent overfitting, was used to represent each unsupervised signature as a linear combination of known signatures (requiring cosine similarity > 0.8). We excluded signatures associated with DNA repair and chemotherapy from candidate signatures as they were not relevant to this study. The algorithm then refits the activity matrix H using the established COSMIC signature matrix W. The values in the refitted H matrix are constrained to non-negative integers, representing the estimated number of mutations attributed to each signature. Specifically, the algorithm first scales the continuous NNLS estimates so that their sum matches the exact total number of mutations in the sample. The scaled values are then rounded down to obtain initial integer counts. Any remaining mutations are distributed to the signatures with the largest fractional parts using the largest remainder method, ensuring that the sum of assigned mutations exactly equals the total observed mutation count. The mutation burden defined as number of mutations per megabase (mut/Mb) was calculated by assuming that an average of 39 Mb of the exome has sufficient coverage, based on UKB sequencing protocols [1].

### 2.5. GWAS Data Collection

We downloaded published GWAS summary statistics (v1.0.e114) from the GWAS Catalog [32] at https://www.ebi.ac.uk/gwas/ (accessed on 30 June 2025). The reference and variant alleles of each association were annotated using dbSNP [33] (v151, GRCh38), which was downloaded from the NCBI FTP server at https://ftp.ncbi.nlm.nih.gov/snp/organisms/ (accessed on 27 October 2022). These variants were subsequently categorized into six substitution types (C>A, C>G, C>T, T>A, T>C, and T>G) and other categories (e.g., insertions and deletions). To reduce the error in calculating the percentage of C>A mutations, only phenotypes with at least 50 associated loci were retained for further analysis. Consequently, a total of 382,636 loci associated with 1668 phenotypes were included in the final analysis.

Next, we manually selected phenotypes that exhibited a strong positive correlation with smoking based on the following criteria: (1) include phenotypes that directly define smoking status (e.g., “ever vs. never smokers”) or quantify smoking intensity and frequency (e.g., “cigarettes smoked per day”); (2) include respiratory diseases strongly associated with smoking, including lung adenocarcinoma, chronic obstructive pulmonary disease (COPD), pulmonary fibrosis, etc.; (3) exclude infectious respiratory diseases (e.g., COVID-19); and (4) exclude phenotypes that had already been adjusted for smoking status. By doing so, 24 phenotypes met these criteria (Supplementary Table S5).

### 2.6. Statistical Analysis

Correlations between continuous variables were assessed using the two-sided Spearman’s rank correlation. The difference between two groups of continuous variables was evaluated using the two-sided Wilcoxon rank-sum test. Furthermore, multiple linear regression models were used to evaluate the contribution of phenotypes to either the total mutation burden or the activity of specific mutational signatures, providing coefficients, 95% CIs, and *p*-values. The following UKB phenotype fields were used as covariates: age (field 21003), sex (field 31), smoking status (field 20116), mean sequencing depth (calculated based on WES data), WES data release batch (field 32050), and the top 10 genomic principal components (PCs, field 22009). All statistical analyses were performed using the Python packages SciPy [29] (v1.15.3) and statsmodels [34] (v0.14.4).

## 3. Results

### 3.1. The Frequency-Dependent Spectral Characteristics of Germline and Somatic Mutations

**Figure 1:**
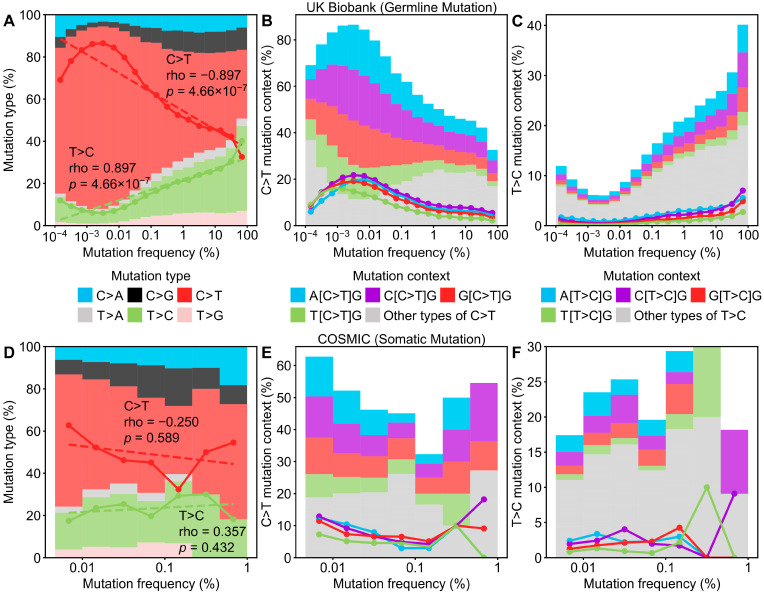
The frequency dependence of mutational spectra in germline vs. somatic mutations. (**A**–**C**) Analysis of germline mutations from UK Biobank (UKB). (**D**–**F**) Analysis of somatic mutations from the COSMIC database, sharing the same color legends with (**A**–**C**), respectively. (**A**,**D**) Stacked bar plots showing the relationship between the mutation frequency and the percentage of the six base substitution types (C>A, C>G, C>T, T>A, T>C, T>G). Mutation frequency is defined as the relative percentage of individuals carrying a specific variant out of the total cohort size. Mutations are grouped into logarithmically equidistant bins (10^1/3^) based on frequency, and the proportion of the six substitution types is calculated for mutations within each bin. For example, the first stacked bar plot on the left in (**A**) represents the percentage of the six substitution types (summing to 100%) among all mutations with a frequency >0.0001% and ≤0.0001 × 10^1/3^%. The percentages of C>T (red) and T>C (green) mutations are additionally displayed as line plots (solid lines), which share the same color legends as the bar plots. The linear regression (dashed line) and Spearman’s rank correlation coefficient (rho) and *p*-value for the correlation between the percentage of C>T and T>C mutations and the mutation frequency are shown. (**B**,**E**) Stacked bar plots showing the percentage of C>T mutations within various trinucleotide contexts, plotted against the mutation frequency. The percentages of the four mutations with a downstream guanine (A[C>T]G, C[C>T]G, G[C>T]G, and T[C>T]G) are additionally displayed as line plots (solid lines), which share the same color legends as the bar plots. (**C**,**F**) Stacked bar plots showing the percentage of T>C mutations within various trinucleotide contexts, plotted against the mutation frequency. The percentages of the four mutations with a downstream guanine (A[T>C]G, C[T>C]G, G[T>C]G, and T[T>C]G) are additionally displayed as line plots (solid lines), which share the same color legends as the bar plots. The x-axis is represented on a logarithmic scale.

Germline mutations are predominantly shaped by long-term evolutionary forces, population history, ancestry, and genetic drift. Somatic mutations, due to their relatively random distribution across the genome, are expected to recur at the same genomic position with a low frequency. Consequently, it is essential to distinguish between the full spectrum of mutations and rare mutations. Low-frequency germline mutations are more likely to represent recent events and are thus more susceptible to harboring de novo somatic mutations. Therefore, we compared the relationship between the spectral characteristics and the number of occurrences for germline mutations derived from blood WES (from UKB) and somatic mutations in hematopoietic and lymphoid tissues (from the COSMIC). The result demonstrates that these two classes of mutations demonstrated markedly different frequency dependencies (Figure 1).

For germline mutations, we observed a strong correlation between the mutation frequency and the proportion of certain mutation type. Specifically, the relative abundance of C>T (G>A) transitions showed a significant negative correlation with mutation frequency (Figure 1A, Spearman’s ρ = −0.897, *p* = 4.66 × 10^−7^). For relatively rare mutations (e.g., frequency <0.01%), C>T mutations constituted nearly 80% of the total. A closer examination of the trinucleotide context of C>T mutations revealed a higher proportion of the four contexts with a downstream guanine (NpCpG) (Figure 1B). Mutations arising from the deamination of 5-methylcytosine (5-mC) at CpG dinucleotides, which have a high spontaneous mutation rate, are recognized as an age-related “clock-like” signature in somatic mutation studies [35,36]. The C>T mutation dominance at low frequencies is biologically expected, as mutations caused by this deamination occur at a continuous, clock-like rate across populations, continuously generating a vast pool of unique, low-frequency mutations. Conversely, T>C (A>G) transitions represented the second most dominant mutation type, and their relative abundance was significantly positively correlated with mutation frequency (Figure 1A, Spearman’s ρ = 0.897, *p* = 4.66 × 10^−7^), eventually surpassing C>T mutations in abundance at the high-frequency end of the curve. Similarly, the four mutational contexts with a downstream guanine were the primary constituents of T>C mutations (Figure 1C). This may be an artifact resulting from differences between the human reference genome and the common genotype in the UK population, as a T>C mutation for most individuals can be interpreted as a C>T mutation for a minority.

We further investigated blood WES data from TCGA and WGS of blood-derived lymphoblastoid cell lines (LCLs) from Phases 1 and 3 of the 1000 Genomes Project. As a result, similar features were observed (Supplementary Figure S1). Namely, C>T and T>C transitions remained the two most abundant mutation types. And the proportion of C>T mutations significantly decreased as mutation frequency increased, whereas the proportion of T>C mutations increased (Supplementary Figure S1A,D,G). At the trinucleotide context level, C>T mutations with a downstream guanine exhibited more changes (Supplementary Figure S1B,E,H). This suggests that this pattern is independent of population, sequencing technology, and reference genome version. Moreover, this phenomenon remained robust under different germline variant filtering criteria (Section 2.2, Supplementary Figure S2). For instance, the frequency-dependent trend holds whether applying alternative combinations of sequencing depth and VAF thresholds (Supplementary Figure S2B, DP ≥ 10, 0.422 ≤ VAF ≤ 0.54 or VAF ≥ 0.9), sequencing depth and variant allele depth (Supplementary Figure S2D, DP ≥ 20, AD ≥ 5), or statistical tests for heterozygosity (Supplementary Figure S2E, DP ≥ 10, VAF ≥ 0.2, binomial test *p* ≥ 1 × 10^−6^). It should be noted that we have employed a stringent filtering strategy that yields a minimal number of variants (Supplementary Figure S2A). Using another common filtering method like the binomial test would result in a larger set of retained variants (Supplementary Figure S2C, DP ≥ 10, VAF ≥ 0.2, binomial test *p* ≥ 1 × 10^−6^). Interestingly, for mutations with low frequency, there was an abnormal decrease in the proportion of C>T mutations, accompanied by an increase in the proportion of mutation types dominated by C>A (G>T) transversions. This increase was more pronounced under lenient filtering criteria, where C>A transversions could even surpass T>C mutations, the original second-most abundant type (Supplementary Figure S2F, DP ≥ 10, AD ≥ 3). This suggests that rare variants may be contaminated with somatic mutations arising from other etiological factors. For instance, polycyclic aromatic hydrocarbons in tobacco smoke can form adducts with bases in CpG islands, a known cause of C>A transversions in smokers’ genomes [37,38].

In contrast, we did not observe such clear patterns among somatic mutations (Figure 1D–F). Although C>T and T>C mutations still represented the two most dominant mutation types, their relative abundances showed no statistically significant correlation with their frequencies in the COSMIC database (Figure 1D; C>T: ρ = −0.250, *p* = 0.589; T>C: ρ = 0.357, *p* = 0.432). The entire mutational spectrum appeared more random and noisier across different frequency bins. Likewise, at the trinucleotide context level, the distribution of somatic mutations did not exhibit any uniform regularity with respect to frequency (Figure 1E,F). As statistical power is affected by the number of data points, we repeated the experiment with different logarithmic scale bin sizes. The results confirmed that the above statistical outcomes are not influenced by bin size. The significant changes in the UKB germline mutation spectrum remained observable with larger bin sizes and fewer data points (Supplementary Figure S3A), while the non-significant changes in the COSMIC somatic mutation spectrum remained non-significant even with smaller bin sizes and more data points (Supplementary Figure S3B). These findings indicate that somatic mutations have a stable frequency distribution, which more closely resembles that of low-frequency germline mutations. This offers a new perspective on how mutational processes at the levels of natural selection and somatic development shape the evolution of the human genome.

### 3.2. Mutational Signatures Reveal Contextual Features of Germline Mutations

**Figure 2:**
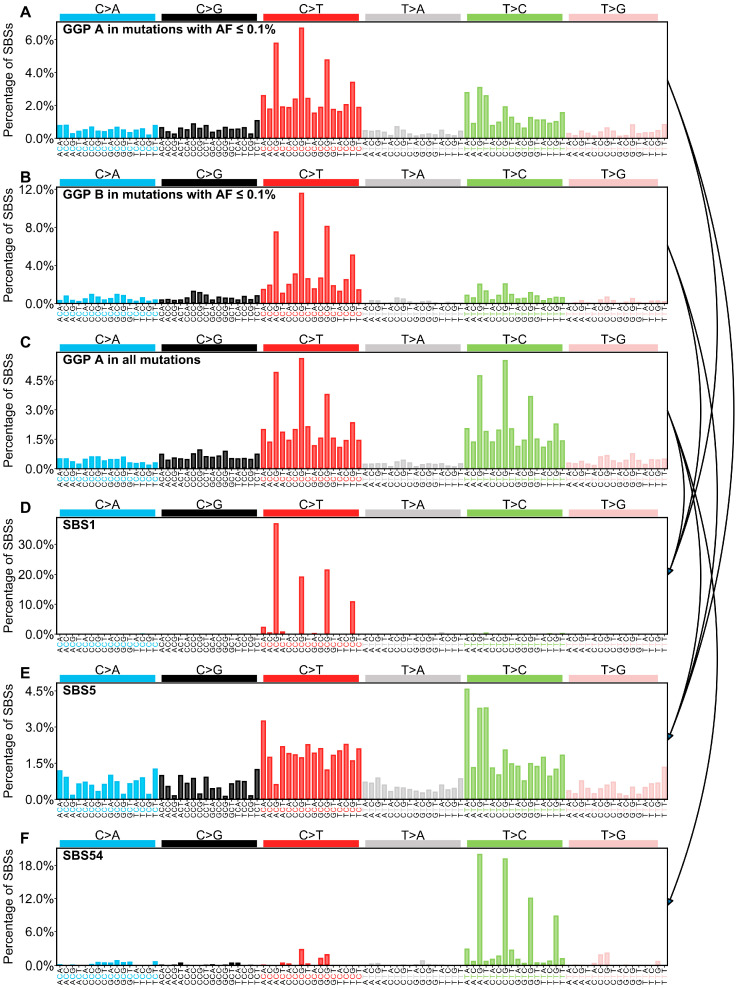
Germline Genome Patterns (GGPs) and their matching to known COSMIC signatures. (**A**,**B**) Two unsupervised GGPs extracted from mutations with allele frequency (AF) ≤ 0.1%. (**C**) One unsupervised GGP extracted from all mutations. (**D**–**F**) Reference COSMIC mutational signatures corresponding to the patterns in (**A**–**C**). The x-axis for all panels displays the 96 trinucleotide contexts of single-base substitutions, which are defined by six substitution types (C>A, C>G, C>T, T>A, T>C, T>G, shown in distinct colors) and their 5′ and 3′ flanking nucleotides. The y-axis represents the percentage of each context. For example, the first bar on the left (under the blue C>A category) represents the percentage of the ACA trinucleotide context mutating to AAA. Arrows connecting the panels (e.g., (**A**,**D**) and (**C**,**F**), respectively) indicate the relationship between the unsupervised GGPs and reference signatures, as determined by non-negative least squares optimization.

To investigate the somatic-like features embedded within the germline mutation spectrum, we analyzed the SBS signatures in all variants and subsets of rare variants using the Germline Genome Pattern (GGP) analysis (Figure 2). In our previous studies [20,39], GGP successfully identified clinically relevant signatures in cancer and COVID-19 patients. For rare variants, two unsupervised GGPs were identified (Figure 2A,B, Supplementary Tables S1 and S2). However, all could be explained by different proportions of classic somatic mutational signatures, namely SBS1 and SBS5. SBS1 [35] displays a high frequency of C>T transitions within NpCpG contexts (Figure 2D) and SBS5 [40] contains broad components involving nearly all types of SBSs (Figure 2E). Although SBS5 was identified early [40], the mutational process it represents remains unclear, with only statistical associations to aging, smoking, and deficiencies in nucleotide excision repair [11]. The detection of a similar signature in the germline genome suggests that SBS5 may represent an average mutational pattern arising from various mutational processes with weak base specificity.

For the entire set of mutations, only a single GGP was extracted (Figure 2C, Supplementary Tables S3 and S4). This is likely because the general background genomic pattern of the population is dominant, masking the effects of other mutational etiologies. This single signature could be decomposed into three COSMIC SBS signatures: SBS1, SBS5, and SBS54. SBS54 is characterized by T>C mutations in NTG contexts (Figure 2F) and is often considered an artifact caused by germline mutations in somatic signature studies [11]. We hypothesize that this may potentially reflect an artifact resulting from inconsistencies between the reference genome and the prevalent genotype in the UK population, though further population-genetics studies are required to confirm this. Given the lower proportion of T>C mutations among rare variants, the absence of SBS54 in rare variants is then reasonable. To ensure the robustness of our signature extraction, we applied the GGP analysis across a range of allele frequency cutoffs (from AF ≤ 10% to AF ≤ 0.01%). As shown in Supplementary Figure S4A, the somatic-like signatures SBS1 and SBS5 were consistently identified across all thresholds, while the germline-associated signature SBS54 was only present in the entire variant set and disappeared in rare variant subsets. In addition to the current filtering strategies (DP ≥ 20, 0.422 ≤ VAF ≤ 0.54 or VAF ≥ 0.9), we applied five alternative filtering strategies (Supplementary Figure S4B). The detection of SBS1 and SBS5 remained consistent across all filtering strategies for both all mutations and mutations with AF ≤ 0.1%. Although some other signatures with unknown etiology (e.g., SBS43) can be detected under relatively relaxed filtering conditions (e.g., DP ≥ 20 and AD ≥ 5), these findings are not observed under all the other filtering strategies. Notably, SBS54 is consistently detected in the all-mutation data across all filtering conditions, but is not detected in mutation subsets with AF ≤ 0.1%.

### 3.3. Germline Mutation Burden Is Associated with Mutagenic Etiologies

To evaluate the contamination of somatic mutations in germline mutation calls, we used a multiple linear regression model to explore the relationships between germline mutation burden and age, sex, and smoking status. Additionally, to account for potential technical confounders and residual population structure, mean sequencing depth, sequencing release batch, and the top 10 genomic PCs were used as covariates. To eliminate confounding effects of ethnicity, only individuals of White ethnicity (self-reported ethnicity, UKB field 21000), who constitute 94.1% of the UKB WES cohort, were included. Furthermore, we restricted the analysis to autosomal mutation burden to avoid sex-related biases. The basic statistics for the variables are presented in Table 1.

**Figure 3:**
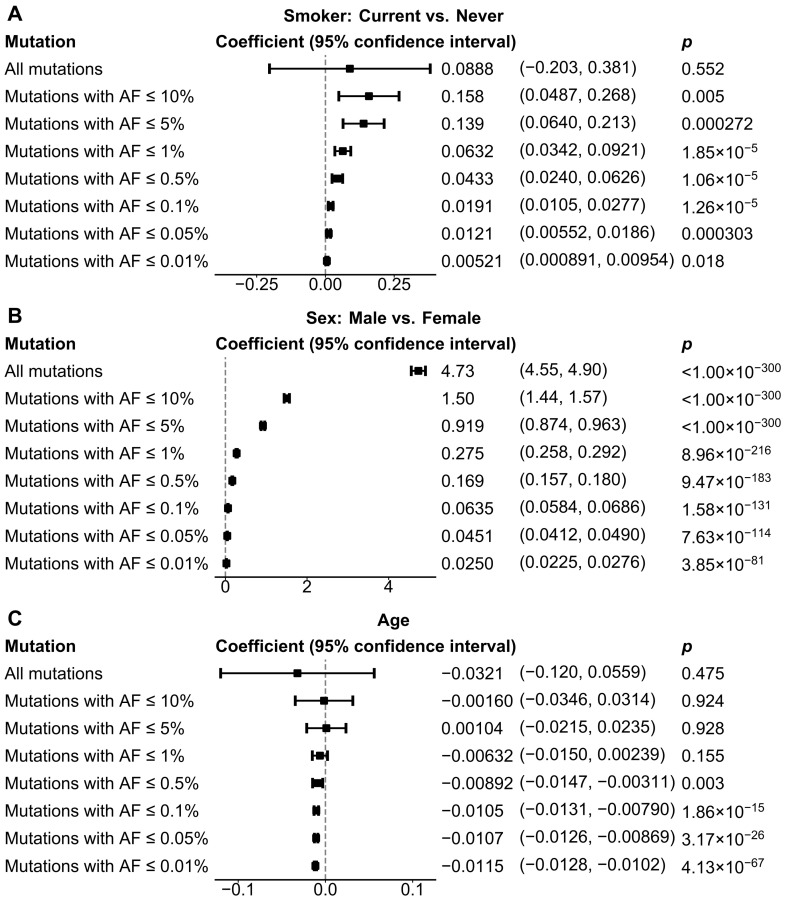
Multivariable analysis of mutagenic factors influencing germline mutation burdens. This figure displays forest plots from multivariable linear regression models, examining the associations of total germline mutation burden with (**A**) smoking status, (**B**) sex, and (**C**) age under different allele frequency (AF) thresholds. The square points represent the estimates of the regression coefficients, and the horizontal lines indicate the 95% confidence intervals (CIs). For smoking status, “never smoking” was used as the reference group, while “female” is the reference for the sex variable. The coefficients, 95% CIs, and *p*-values for each variable are listed to the right of each panel. Please refer to Table 1 for the detailed statistics of mutation burdens corresponding to each AF threshold. The number of individuals included in the multivariable linear regression analysis was 440,759.

Although the association with current smoking status was not statistically significant for the total germline mutation burden (Figure 3A, *p* = 0.552), the association became statistically significant across all rare variant subsets (AF ≤ 10% to 0.01%), with current smokers showing a higher burden than never smokers. This suggests that smoking-induced mutational damage is primarily masked by unrelated germline mutations and could be detected in the rare variant spectrum. More strikingly, males were found to accumulate 4.73 more mut/Mb than females (Figure 3B, 95% confidence intervals [CIs]: 4.55–4.90, *p* < 1 × 10^−300^), possibly reflecting different rates of mutation accumulation due to differences in DNA repair and hormone levels between sexes [41,42]. As the median tumor mutation burden for many types of cancer is less than 4 mut/Mb [43,44], these findings could present a significant impact.

A paradoxical observation was a significant negative correlation between age and mutation burden (Figure 3C). For the total variant set, this correlation was not significant. However, for rare variants, a significant negative correlation emerged at low AFs (≤0.5%). This contradicts the generally accepted positive correlation between age and mutational burden in somatic studies [8,11]. We hypothesize that this is an artifact of our variant filtering strategy: as individuals age, they accumulate more low-VAF somatic mutations (CHIP). Our stringent filtering inevitably removes a greater proportion of variants in older individuals compared to younger ones, creating an artifactual negative association. Therefore, when investigating the relationship between the burden of mutations with lower AF (from ≤0.5% to 0.01%) and age, an increasingly significant negative correlation could be observed (*p* = 0.003, 1.86 × 10^−15^, 3.17 × 10^−26^, and 4.13 × 10^−67^, respectively). This would also imply that our estimates of the effects of other mutagenic factors are conservative, and their actual impact on mutation burden may be more severe.

We also analyzed the burdens of mutations attributed to SBS1 and SBS5. For SBS1 (Supplementary Figure S5), the association with smoking was not significant across any allele frequency threshold. Given that smoking primarily induces C>A mutations [38,45], which is distinct from the predominant component of SBS1 (C>T), it is reasonable to expect that the burden of SBS1 is not correlated with smoking. Similar to the total burden, the sex difference remained highly significant across all subsets, and the negative correlation with age became significant for rare variants. For SBS5 (Supplementary Figure S6), all the results were similar to the total burden. Specifically, the smoking association was not significant for the total set but became significant for all rare variant subsets. The sex difference was consistently significant, while the negative correlation with age was observed at low frequencies.

When considering alternative filtering strategies, although the effect sizes varied, the associations between mutation burden and smoking/sex remained significant (Supplementary Figures S7A,B and S8A,B), and the negative correlation with age became significant for mutations with AF ≤ 0.1% (Supplementary Figures S7C and S8C). Notably, when the filtering strategies are relaxed (e.g., DP ≥ 10 and AD ≥ 3), a significant correlation between smoking and mutational burden can be observed even in the all-mutation data (Supplementary Figure S7A, minimum *p* = 3.75 × 10^−14^), suggesting that relaxed filtering strategies allow more smoking-induced somatic mutation artifacts to be retained. In summary, our stratified analysis reveals that while common germline variants are largely unaffected, the rare variant spectrum, which is more susceptible to somatic contamination, carries a strong signature of smoking, sex, and age.

### 3.4. Spurious Germline Mutations Highlight the Risk of Bias in GWAS Results

Smoking, a major risk factor for multiple cancers and respiratory diseases, is widely recognized for inducing a higher proportion of C>A transversions in somatic genomes [38,45]. Our multivariable regression analysis demonstrated that smoking generates a statistically significant increase in the mutation burden within the “germline” mutation calls (e.g., an estimated increase of 0.158 mut/Mb for mutations with AF ≤ 10% in current smokers). Thus, we wondered whether GWASs of smoking-related phenotypes have been affected. Specifically, because smokers are biologically predisposed to developing both higher somatic mutation burdens (such as CHIP) and smoking-related diseases, these acquired somatic variants become statistically enriched in the patient cohort. GWAS models may then misinterpret these enriched somatic signals as inherited genetic risk factors, thereby creating a fallacious causal link.

**Figure 4:**
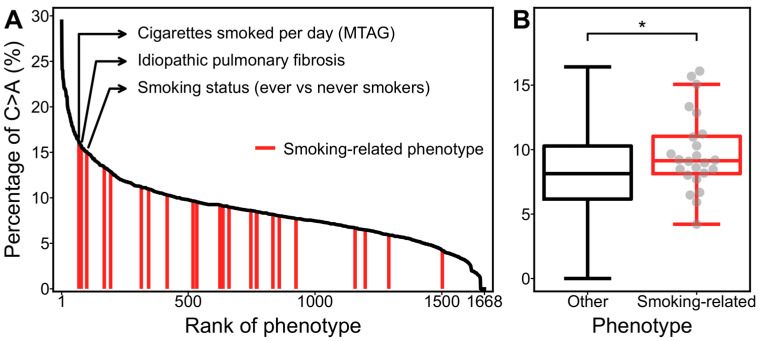
The loci associated with smoking-related phenotypes exhibit a higher proportion of C>A mutations. (**A**) Associated risk loci for 1668 phenotypes are sorted in descending order by the percentage of C>A mutations. Phenotypes manually annotated as smoking-related are marked by red vertical lines. Arrows highlight the 3 smoking-related phenotypes with the highest C>A mutation percentages. (**B**) A box plot comparing the percentage of C>A mutations between the smoking-related phenotype group (red box, *n* = 24) and all other phenotypes (black box, *n* = 1644). The center line of each box represents the median; the lower and upper hinges of the box correspond to the first and third quartiles, respectively. The whiskers extend to the furthest data points within 1.5 times the interquartile range (IQR). The dots in the smoking-related group represent the values for each individual phenotype. * *p* < 0.05 by two-sided Wilcoxon rank-sum test.

Therefore, we collected 382,636 association loci for 1668 phenotypes and calculated the proportion of C>A mutations at the associated loci for each phenotype (Supplementary Table S5). Among these, we focused on 24 phenotypes due to their strong positive correlation with smoking. Notably, 18 (75%) of these smoking-related phenotypes, such as smoking quantity (cigarettes per day, based on multi-trait analysis of GWASs [MTAG]), idiopathic pulmonary fibrosis, and smoking status (ever vs. never smoker), ranked in the top 50% for their percentage of associated C>A mutations (Figure 4A). Statistical analysis revealed that the percentage of C>A transversions among mutations associated with smoking-related phenotypes was significantly higher than in those associated with other phenotypes (Figure 4B, *p* = 0.0469, two-sided Wilcoxon rank-sum test). Our findings suggest that artifacts from smoking-induced germline mutations may have introduced bias in previously published GWAS results. These mutations and the smoking-associated phenotypes are likely common consequences of smoking, creating a fallacious causal link from germline polymorphism to phenotype that could misdirect subsequent research based on these findings.

## 4. Discussion

De novo germline mutations in a new generation originate from somatic mutations within the parental germ cells. In this study, using mutational signature analysis and cosine similarity, we observed that the mutational profile of low-frequency germline variants is similar to the characteristics of somatic mutations found in cancer genomics. We successfully decomposed the classic somatic mutational signatures, SBS1 and SBS5, from the spectrum of germline mutations. As “clock-like” signatures, SBS1 and SBS5 are detectable in nearly all tumor types and even in normal tissues [11,46]. The striking similarity between germline and somatic mutational signatures provides compelling genomic evidence of widespread somatic admixture within blood-derived datasets, suggesting a unified principle underlying species evolution at the population level and somatic clonal evolution at the individual level.

Previous research has established that CHIP is a common age-related phenomenon, and its associated mutations (e.g., in genes such as DNMT3A and TET2) are risk factors for cardiovascular disease and hematologic malignancies [18,47,48]. However, the influence of CHIP signals has been rarely considered in GWASs. Our analysis reveals theoretically implausible yet significant associations between germline mutation burden and age, sex, and smoking status, particularly for rare mutations. These findings raise concerns about the potential impact of residual somatic contamination on the current paradigm of genetic association studies and Mendelian randomization studies by highlighting the potential for CHIP-mediated confounding effects and the risk of inverting cause and effect. For instance, smoking induces somatic mutations in hematopoietic stem cells and is also a cause of smoking-related diseases such as COPD and lung cancer. This can lead to spurious associations in GWAS between smoking-induced pseudo “germline” variants and the disease. In our prior work [20], we identified seven cancer GGPs (CGGPs) from germline genomes in TCGA. One of these, CGGP E, exhibited a robust and significant positive correlation with the somatic mutational signature SBS4, a well-established signature associated with tobacco smoking [37], independent of cancer type, sex, and age. Assuming that the germline genome will not change afterbirth, we initially interpreted this as evidence of the germline genome’s influence on an individual’s susceptibility to smoking-related diseases. From our current perspective, however, these findings suggest that smoking induces a widespread somatic mutation burden in the hematopoietic system, which systematically contaminates ‘germline’ variant calls derived from blood DNA, with both CGGP E and SBS4 being joint consequences of tobacco exposure. Although the unsupervised GGPs matched known COSMIC signatures (SBS1, SBS5), it is important to interpret this with caution. Similarity in mutational spectrum does not necessarily imply identical biological etiology. SBS1 and SBS5 are “clock-like” signatures that may reflect general DNA damage and repair processes rather than specific environmental exposures. Thus, their presence in germline data suggests a shared underlying mutational process, but not necessarily direct somatic contamination from a specific source. These computational alignments warrant future experimental validation.

While our statistical models provide population-level estimates of this somatic admixture (e.g., the significant mutation burden disparities linked to sex and smoking), precisely defining the absolute size of the contamination for any particular pathology remains challenging. A seemingly contradictory finding in this study is the negative correlation between age and the mutation burden after filtering. We propose that this is driven by a pronounced filtering bias tied to the biology of aging. As age increases, the number of CHIP mutations rises substantially. Consequently, older individuals possess a higher number of somatic variants with VAFs approaching our filtering boundaries. When rigid, VAF-based and depth-based bioinformatics filters are applied to remove these somatic contaminants, the algorithms inevitably trigger more aggressively in older samples. This strict filtering acts as a double-edged sword. While attempting to purge the high volume of CHIP mutations in older individuals, it inadvertently excludes a proportionally greater number of true, rare germline variants due to statistical noise. Because we have adjusted for sequencing depth as a covariate in our models, this correlation may not be caused by the variations in sequencing coverage. Furthermore, this observation implies that the estimated impact of other factors, such as smoking, may also be severely underestimated. Our stringent VAF and depth filtering strategies were designed to aggressively eliminate obvious low-VAF variants. Therefore, the somatic admixture we still observe represents merely the “tip of the iceberg”, the cryptic somatic variants whose VAFs overlap with germline thresholds. Estimating the absolute burden is confounded by the ubiquitous nature of clock-like signature SBS5. Because the precise biological etiology of SBS5 remains partially undefined, it is exceedingly difficult to cleanly partition true germline variants from somatic noise based solely on spectral characteristics. Despite these challenges in absolute quantification, the systemic presence and statistical impact of this somatic contamination in large-scale datasets are undeniable and require rigorous methodological attention.

This study reveals the stubborn contamination of somatic mutations within germline genomic data from the large-scale UKB WES cohort. The core finding of this study is that the rare variant pool in blood-derived genomic data is not purely germline, but is systematically admixed with somatic mutations from clonal hematopoiesis. Because somatic mutagenesis is driven by factors like age, sex, and smoking, these contaminated rare variants inherently carry the statistical footprint of these exposures. When GWAS models test for associations between genetic variants and phenotypes driven by the same exposures (e.g., smoking and COPD), the shared etiology between the variant’s origin (smoking-induced somatic mutation) and the phenotype (smoking-induced disease) creates a confounding loop, elevating the risk of spurious associations. We should recognize the limitations of current filtering strategies and reconsider the definition and operational standards of “germline” in studies utilizing blood-derived DNA. For example, machine learning models could be developed to more accurately estimate the probability of a variant being of germline or somatic origin by integrating multi-dimensional features, including VAF, trinucleotide context, sequencing depth, strand bias, and known CHIP drivers. Moreover, incorporating CHIP status and exposure to mutagens like tobacco smoke as covariates in statistical models may be an effective strategy to mitigate their confounding effects in association analyses.

Nevertheless, this study has its limitations. We utilized the COSMIC database as a robust reference for somatic mutations. These profiles are derived from hematopoietic and lymphoid malignancies. The mutational landscape may not fully mirror the dynamics of somatic mutations in normal aging blood or early-stage CHIP. Future studies utilizing ultra-deep sequencing of non-malignant blood samples are required to establish a more precise baseline for CHIP-specific mutational signatures. Furthermore, our analysis was predominantly based on the White population from the UK, and validation in large-scale cohorts of other ancestries is lacking. The GWAS loci we used relied on published summary statistics, which were not performed under the same population and analysis pipeline, thus could introduce confounding effects on our result. While our findings provide evidence of somatic mutational contamination in germline datasets, this effect is a statistical observation, and we cannot pinpoint exactly which specific loci are contaminated. A long-term, longitudinal study with repeated sequencing is warranted to more precisely assess the dynamics of the germline genome over an individual’s lifespan.

## 5. Conclusions

In conclusion, this study introduces a novel approach by applying mutational signature analysis, traditionally utilized in cancer genomics, to systematically evaluate large-scale germline cohorts. Our study demonstrates that current variant filtering strategies based on VAF and sequencing depth are insufficient to effectively purge somatic mutational contamination from large-scale blood-derived germline genomic datasets. This widespread somatic admixture introduces substantial confounding effects, as evidenced by the spurious associations of rare “germline” variants with mutagenic factors like age, sex, and smoking status. This phenomenon highlights the potential biases introduced into published GWAS findings. To ensure the accuracy, reproducibility, and biological interpretability of large-scale association studies, future research should adopt more stringent and multi-dimensional variant filtering approaches to strictly distinguish true germline variants from somatic contaminants.

## Figures and Tables

**Table 1:** Characteristics and mutation burden of study cohort from UK Biobank.

	Category	Unit	Statistics
*n*	-	-	440,759
Age, mean (std)	-	year	56.5 (8.09)
Sex, *n* (%)	Female	-	239,148 (54.3)
	Male	-	201,611 (45.7)
Smoking status, *n* (%)	Never	-	237,749 (53.9)
	Previous	-	156,853 (35.6)
	Current	-	46,157 (10.5)
Total burden, mean (std)	Total mutation	mut/Mb	809 (104)
	Mutation with AF ≤ 10%	mut/Mb	142 (36.7)
	Mutation with AF ≤ 5%	mut/Mb	80.7 (23.0)
	Mutation with AF ≤ 1%	mut/Mb	25.5 (7.54)
	Mutation with AF ≤ 0.5%	mut/Mb	16.9 (4.86)
	Mutation with AF ≤ 0.1%	mut/Mb	7.79 (2.12)
	Mutation with AF ≤ 0.05%	mut/Mb	6.08 (1.60)
	Mutation with AF ≤ 0.01%	mut/Mb	3.69 (0.942)
SBS1 burden, mean (std)	Total mutation	mut/Mb	120 (13.0)
	Mutation with AF ≤ 10%	mut/Mb	28.3 (5.25)
	Mutation with AF ≤ 5%	mut/Mb	16.7 (3.36)
	Mutation with AF ≤ 1%	mut/Mb	5.69 (1.22)
	Mutation with AF ≤ 0.5%	mut/Mb	3.90 (0.870)
	Mutation with AF ≤ 0.1%	mut/Mb	1.93 (0.491)
	Mutation with AF ≤ 0.05%	mut/Mb	1.52 (0.400)
	Mutation with AF ≤ 0.01%	mut/Mb	0.891 (0.262)
SBS5 burden, mean (std)	Total mutation	mut/Mb	527 (79.0)
	Mutation with AF ≤ 10%	mut/Mb	113 (31.7)
	Mutation with AF ≤ 5%	mut/Mb	64.0 (19.8)
	Mutation with AF ≤ 1%	mut/Mb	19.9 (6.46)
	Mutation with AF ≤ 0.5%	mut/Mb	12.8 (4.19)
	Mutation with AF ≤ 0.1%	mut/Mb	5.86 (1.73)
	Mutation with AF ≤ 0.05%	mut/Mb	4.56 (1.30)
	Mutation with AF ≤ 0.01%	mut/Mb	2.80 (0.760)
SBS54 burden, mean (std)	Total mutation	mut/Mb	162 (13.8)

Abbreviations: AF, allele frequency; std, standard deviation with delta degree(s) of freedom = 1; mut/Mb, mutations per megabase; SBS, single-base substitution. SBS1, SBS5, and SBS54 refer to specific mutational signatures defined by COSMIC database.

## Data Availability

The data used in this study are available from the UKB under specific restrictions. As the data were accessed under license, they are not publicly available. Access to UK Biobank data can be requested through the standard application process at https://www.ukbiobank.ac.uk/use-our-data/apply-for-access/ (accessed on 20 April 2023). The TCGA germline mutation data are available according to the authorization procedure described on this website: http://isb-cancer-genomics-cloud.readthedocs.io/en/latest/sections/webapp/Gaining-Access-To-Contolled-Access-Data.html (accessed on 9 March 2020). The 1000 Genomes Project data are available from https://ftp.1000genomes.ebi.ac.uk/vol1/ftp/release/ (accessed on 22 November 2022). The frequencies of somatic mutations are available from COSMIC, https://cancer.sanger.ac.uk/cosmic (accessed on 19 January 2024). The GWAS associations are available from GWAS Catalog, https://www.ebi.ac.uk/gwas/ (accessed on 30 June 2025), and the variants can be annotated using dbSNP, https://ftp.ncbi.nlm.nih.gov/snp/organisms/ (accessed on 27 October 2022). All data supporting the findings of this study are included in the article and Supplementary Materials and can be obtained from the corresponding author upon request.

## Supplementary Materials

The following supporting information can be downloaded at: https://www.mdpi.com/article/10.3390/biology15141204/s1, Figure S1: Frequency dependence of germline mutational spectra in various datasets; Figure S2: Robustness analysis of the relationship between germline mutational spectrum and mutation frequency; Figure S3: Frequency dependence of mutational spectra in germline vs. somatic mutations under different binning resolutions; Figure S4: The detection of mutation signatures under different filtering strategies; Figure S5: Multivariable analysis of mutagenic factors influencing SBS1-related germline mutation burdens; Figure S6: Multivariable analysis of mutagenic factors influencing SBS5-related germline mutation burdens; Figure S7: Multivariable analysis of mutagenic factors influencing germline mutation burdens under different filtering strategies; Figure S8: Multivariable analysis of mutagenic factors influencing rare germline mutation burdens under different filtering strategies; Table S1: The selection of k for unsupervised extraction of signatures in rare mutations; Table S2: The unsupervised extracted germline genome patterns (GGPs) in rare mutations; Table S3: The selection of k for unsupervised extraction of signatures in all mutations; Table S4: The unsupervised extracted germline genome pattern (GGP) in all mutations; Table S5: The percentage of mutation types associated with various phenotypes.

## Abbreviations

The following abbreviations are used in this manuscript:

5-mC	5-methylcytosine
AF	allele frequency
CGGP	Cancer Germline Genome Pattern
CH	clonal hematopoiesis
CHIP	clonal hematopoiesis of indeterminate potential
COPD	chronic obstructive pulmonary disease
COSMIC	Catalogue of Somatic Mutations in Cancer
ExWAS	exome-wide association study
GGP	Germline Genome Pattern
GWAS	genome-wide association study
LCL	lymphoblastoid cell line
MTAG	multi-trait analysis of GWAS
mut/Mb	mutations per megabase
OQFE	Original Quality Functional Equivalent
PC	principal component
pQTL	protein quantitative trait loci
SBS	single-base substitution
TCGA	The Cancer Genome Atlas
UKB	UK Biobank
VAF	variant allele frequency
WES	whole-exome sequencing
WGS	whole-genome sequencing
