# Supplementary material for: Evaluating Somatic Mutational Contamination in Large-Scale Germline Genomic Studies
Source: Biology (Basel). 2026 Jul 21;15(14):1204. doi: 10.3390/biology15141204 (PMC13403583; doi:10.3390/biology15141204)
Supplement: Supplementary file 1 [file biology-15-01204-s001.zip › Supplementary Figures.pdf]

Evaluating Somatic Mutational Contamination in Large-Scale Germline Genomic Studies

Xiangwen Ji [1], Xueke Bai [2], Guangda He [2], Kai Yan [3], Edwin Wang [3], Yi-Da Tang [1],

Liang Chen [2,*], and Qinghua Cui [1,4,5,*]

[1]Department of Cardiology and Institute of Vascular Medicine, State Key Laboratory of Vascular Homeostasis and Remodeling, Peking University Third Hospital, 49 Huayuanbei Road, Beijing 100191, China.

[2]National Clinical Research Center for Cardiovascular Diseases, State Key Laboratory of Cardiovascular Disease, Fuwai Hospital, National Center for Cardiovascular Diseases, Chinese Academy of Medical Sciences and Peking Union Medical College, Beijing 100037, China.

[3]Department of Biochemistry and Molecular Biology, Medical Genetics, and Oncology, Cumming School of Medicine, University of Calgary, Calgary, AB T2N 1N4, Canada

[4]School of Sports Medicine, Wuhan Sports University, No. 461 Luoyu Rd. Hongshan District, Wuhan 430079, Hubei Province, China.

[5]Department of Biomedical Informatics, State Key Laboratory of Vascular Homeostasis and Remodeling, School of Basic Medical Sciences, Peking University, 38 Xueyuan Rd, Beijing, 100191, China.

[*]Correspondence: liang.chen9@hotmail.com (L.C.); cuiqinghua@bjmu.edu.cn (Q.C.)

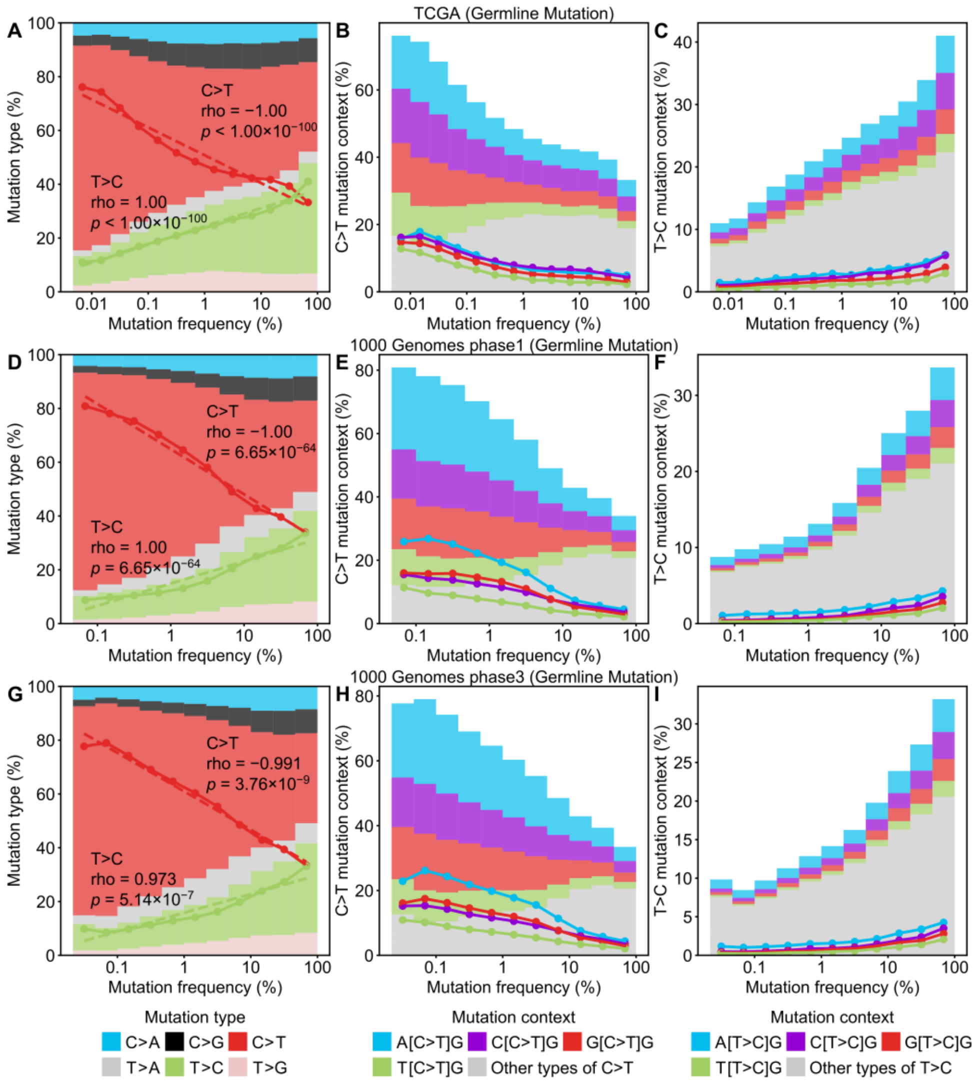

Supplementary Figure S1. The frequency dependence of germline mutational spectra in various datasets. The analysis was performed on germline mutations from (**A-C**) The Cancer Genome Atlas (TCGA), (**D-F**) 1000 Genomes Project Phase 1, and (G-I) 1000 Genomes Project Phase 3. (**A**,**D**,**G**) The stacked bar plots showing the relationship between the percentage of the six base substitution types (C>A, C>G, C>T, T>A, T>C, and T>G) and the mutation frequency. Mutation frequency is defined as the relative percentage of individuals carrying a specific variant out of the total cohort size. The percentages of C>T (red) and T>C (green) mutations are additionally displayed as line plots (solid lines), which share the same color legends as the bar plots. The linear regression (dashed line) and Spearman's rank correlation coefficient (rho) and p-value for the correlation between the percentage of C>T and T>C mutations and the mutation frequency are shown. (**B**,**E**,**H**) The stacked bar plots showing the percentage of C>T mutations within various trinucleotide contexts, plotted against the mutation frequency. The percentages of the four mutations with a downstream guanine (A[C>T]G, C[C>T]G, G[C>T]G, and T[C>T]G) are additionally displayed as line plots (solid lines), which share the same color legends as the bar plots. (**C**,**F**,**I**) The stacked bar plots showing the percentage of T>C mutations within various trinucleotide contexts, plotted against the mutation frequency. The percentages of the four mutations with a downstream guanine (A[T>C]G, C[T>C]G, G[T>C]G, and T[T>C]G) are additionally displayed as line plots (solid lines), which share the same color legends as the bar plots. The x-axis is represented on a logarithmic scale.

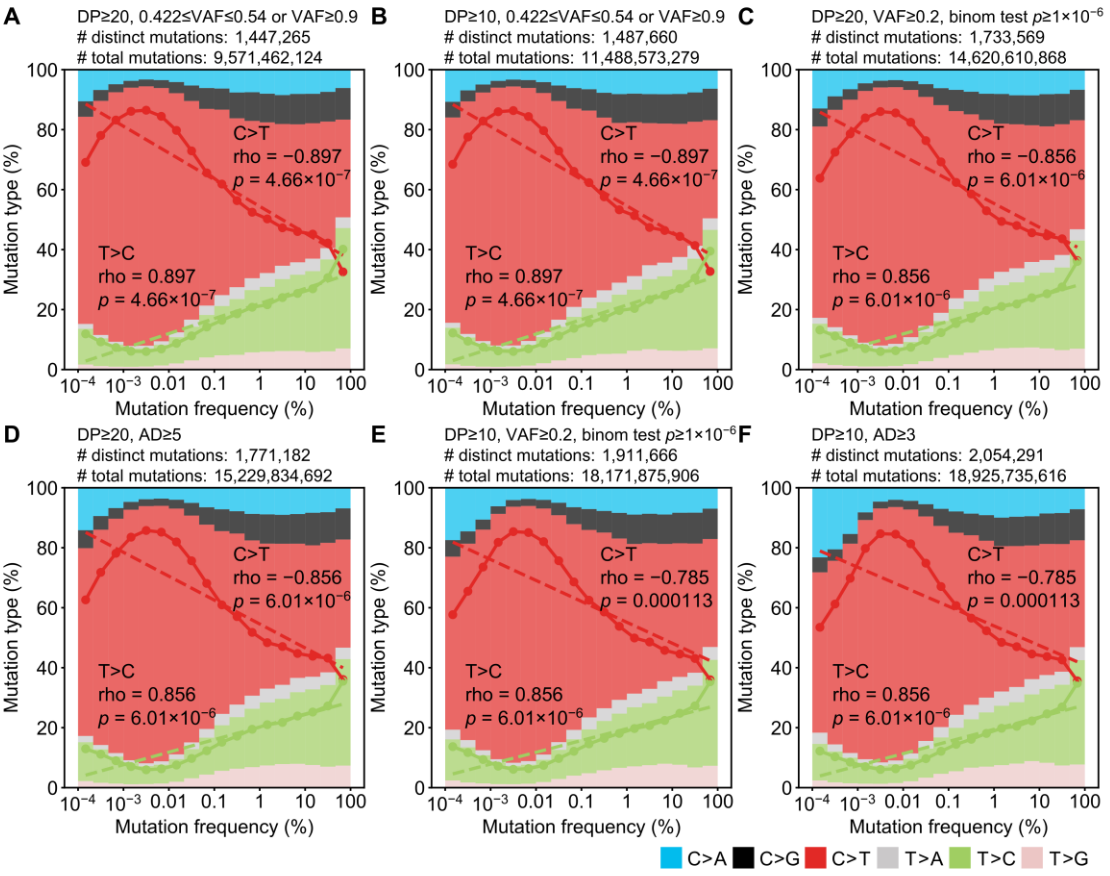

Supplementary Figure S2. Robustness analysis of the relationship between germline mutational spectrum and mutation frequency. (**A**-**F**) Stacked bar plots showing the relationship between the percentage of the six base substitution types (C>A, C>G, C>T, T>A, T>C, and T>G) and the mutation frequency, under six different mutation filtering criteria. Mutation frequency is defined as the relative percentage of individuals carrying a specific variant out of the total cohort size. The percentages of C>T (red) and T>C (green) mutations are additionally displayed as line plots (solid lines), which share the same color legends as the bar plots. The linear regression (dashed line) and Spearman's rank correlation coefficient (rho) and p-value for the correlation between the percentage of C>T and T>C mutations and the mutation frequency are shown. (**A**-**F**) use the same color legend as shown at the bottom. The x-axis is represented on a logarithmic scale. DP: total sequencing depth; VAF: variant allele frequency; AD: variant allele depth; binom test: binomial test between VAF and 50%.

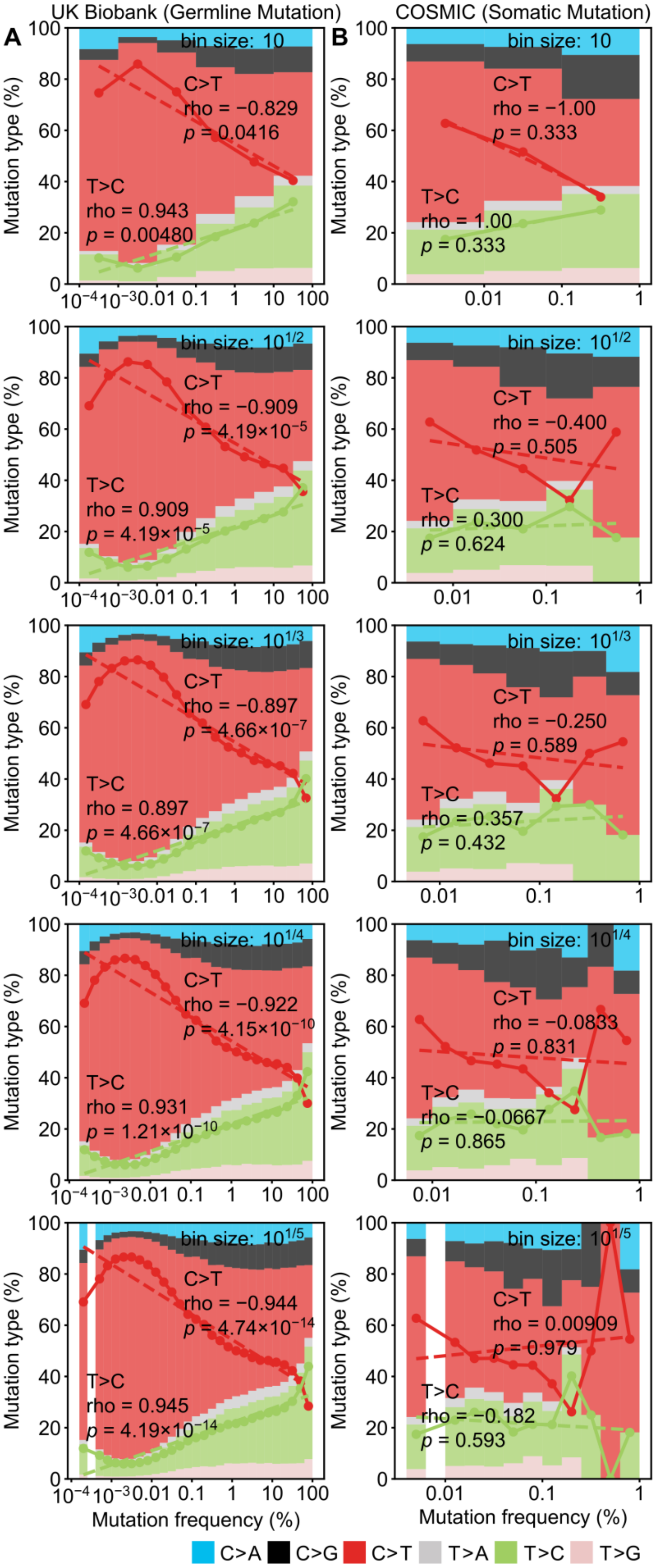
UK Biobank (Germline Mutation)
COSMIC (Somatic Mutation)
A
B
bin size: 10
C>T
rho = −0.829
p = 0.0416
T>C
rho = 0.943
p = 0.00480
C>T
rho = −1.00
p = 0.333
T>C
rho = 1.00
p = 0.333
bin size: 10^1/2
C>T
rho = −0.909
p = 4.19×10^−5
T>C
rho = 0.909
p = 4.19×10^−5
C>T
rho = −0.400
p = 0.505
T>C
rho = 0.300
p = 0.624
bin size: 10^1/3
C>T
rho = −0.897
p = 4.66×10^−7
T>C
rho = 0.897
p = 4.66×10^−7
C>T
rho = −0.250
p = 0.589
T>C
rho = 0.357
p = 0.432
bin size: 10^1/4
C>T
rho = −0.922
p = 4.15×10^−10
T>C
rho = 0.931
p = 1.21×10^−10
C>T
rho = −0.0833
p = 0.831
T>C
rho = −0.0667
p = 0.865
bin size: 10^1/5
C>T
rho = −0.944
p = 4.74×10^−14
T>C
rho = 0.945
p = 4.19×10^−14
C>T
rho = 0.00909
p = 0.979
T>C
rho = −0.182
p = 0.593
Mutation type (%)
Mutation frequency (%)
C>A C>G C>T T>A T>C T>G

Supplementary Figure S3. The frequency dependence of mutational spectra in germline vs. somatic mutations under different binning resolutions. (**A**) Analysis of germline mutations from the UK Biobank. (**B**) Analysis of somatic mutations from the COSMIC database. The stacked bar plots in each panel show the relationship between the percentage of the six base substitution types (C>A, C>G, C>T, T>A, T>C, and T>G) and the mutation frequency. Mutation frequency is defined as the relative percentage of individuals carrying a specific variant out of the total cohort size. Each row displays the results for a different logarithmic scale bin size of mutation frequency. The percentages of C>T (red) and T>C (green) mutations are additionally displayed as line plots (solid lines), which share the same color legends as the bar plots. The linear regression (dashed line) and Spearman's rank correlation coefficient (rho) and p-value for the correlation between the percentage of C>T and T>C mutations and the mutation frequency are shown. All panels use the same color legend as shown at the bottom. The x-axis is represented on a logarithmic scale.

A

	SBS1	SBS5	SBS54
All mutations	✓	✓	✓
Mutations with AF ≤ 10%	✓	✓	✗
Mutations with AF ≤ 5%	✓	✓	✗
Mutations with AF ≤ 1%	✓	✓	✗
Mutations with AF ≤ 0.5%	✓	✓	✗
Mutations with AF ≤ 0.1%	✓	✓	✗
Mutations with AF ≤ 0.05%	✓	✓	✗
Mutations with AF ≤ 0.01%	✓	✓	✗

B

	SBS1	SBS5	SBS8	SBS17b	SBS18	SBS22a	SBS43	SBS54	SBS55	SBS56	
DP≥20, 0.422≤VAF≤0.54 or VAF≥0.9	✓	✓	✗	✗	✗	✗	✗	✓	✗	✗	All
DP≥20, 0.422≤VAF≤0.54 or VAF≥0.9	✓	✓	✗	✗	✗	✗	✗	✗	✗	✗	AF ≤ 0.1%
DP≥10, 0.422≤VAF≤0.54 or VAF≥0.9	✓	✓	✗	✗	✗	✗	✗	✓	✗	✗	All
DP≥10, 0.422≤VAF≤0.54 or VAF≥0.9	✓	✓	✗	✗	✗	✗	✗	✗	✗	✗	AF ≤ 0.1%
DP≥20, VAF≥0.2, binom test $p \geq 1\times10^{-6}$	✓	✓	✗	✗	✗	✗	✗	✓	✗	✗	All
DP≥20, VAF≥0.2, binom test $p \geq 1\times10^{-6}$	✓	✓	✗	✗	✗	✗	✗	✗	✗	✗	AF ≤ 0.1%
DP≥20, AD≥5	✓	✓	✗	✗	✗	✗	✓	✓	✗	✗	All
DP≥20, AD≥5	✓	✓	✗	✗	✗	✗	✗	✗	✗	✗	AF ≤ 0.1%
DP≥10, VAF≥0.2, binom test $p \geq 1\times10^{-6}$	✓	✓	✗	✗	✗	✗	✗	✓	✗	✗	All
DP≥10, VAF≥0.2, binom test $p \geq 1\times10^{-6}$	✓	✓	✓	✓	✓	✗	✗	✗	✓	✓	AF ≤ 0.1%
DP≥10, AD≥3	✓	✓	✗	✗	✗	✗	✓	✓	✗	✗	All
DP≥10, AD≥3	✓	✓	✓	✗	✗	✓	✗	✗	✗	✓	AF ≤ 0.1%

Supplementary Figure S4. The detection of mutation signatures under different filtering strategies. (**A**) Mutation signatures detected in mutations with different allele frequency (AF) thresholds under the original filtering strategy (DP ≥ 20, 0.422 ≤ VAF ≤ 0.54 or VAF ≥ 0.9). Checkmarks indicate detection, whereas crosses indicate non-detection. (**B**) Mutation signatures detected in all mutations and rare mutations under alternative mutation filtering strategies. DP: total sequencing depth; VAF: variant allele frequency; AD: variant allele depth; binom test: binomial test between VAF and 50%.

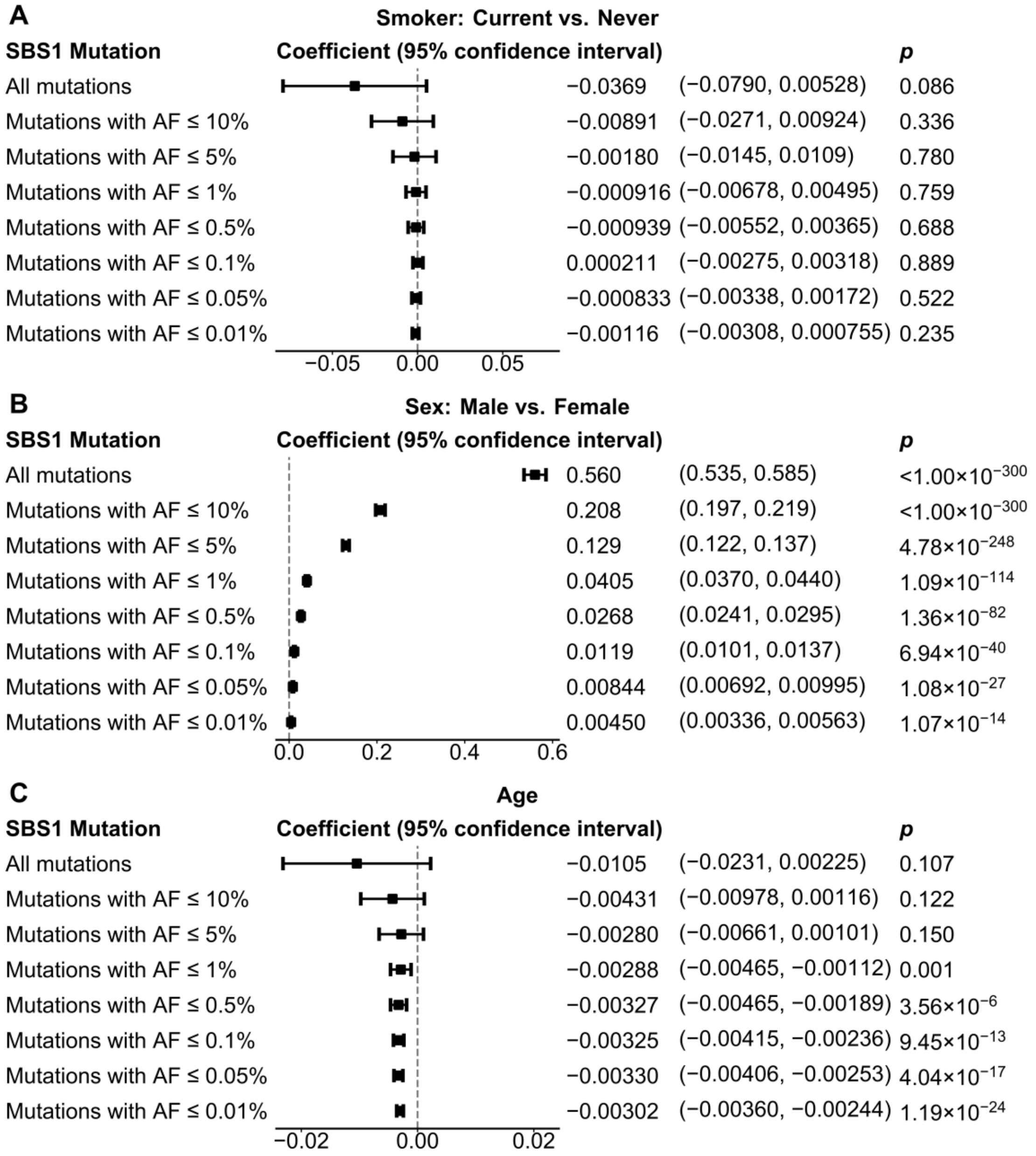

Supplementary Figure S5. Multivariable analysis of mutagenic factors influencing SBS1-related germline mutation burdens. This figure displays forest plots from multivariable linear regression models, examining the associations of SBS1-related germline mutation burden with (**A**) smoking status, (**B**) sex, and (**C**) age under different allele frequency (AF) thresholds. The square points represent the estimates of the regression coefficients, and the horizontal lines indicate the 95% confidence intervals (CIs). For smoking status, "never smoking" was used as the reference group, while "female" is the reference for the sex variable. The coefficients, 95% CIs, and *p*-values for each variable are listed to the right of each panel. Please refer to Table 1 for the detailed statistics of mutation burdens corresponding to each AF threshold. The number of individuals included in the multivariable linear regression analysis was 440,759.

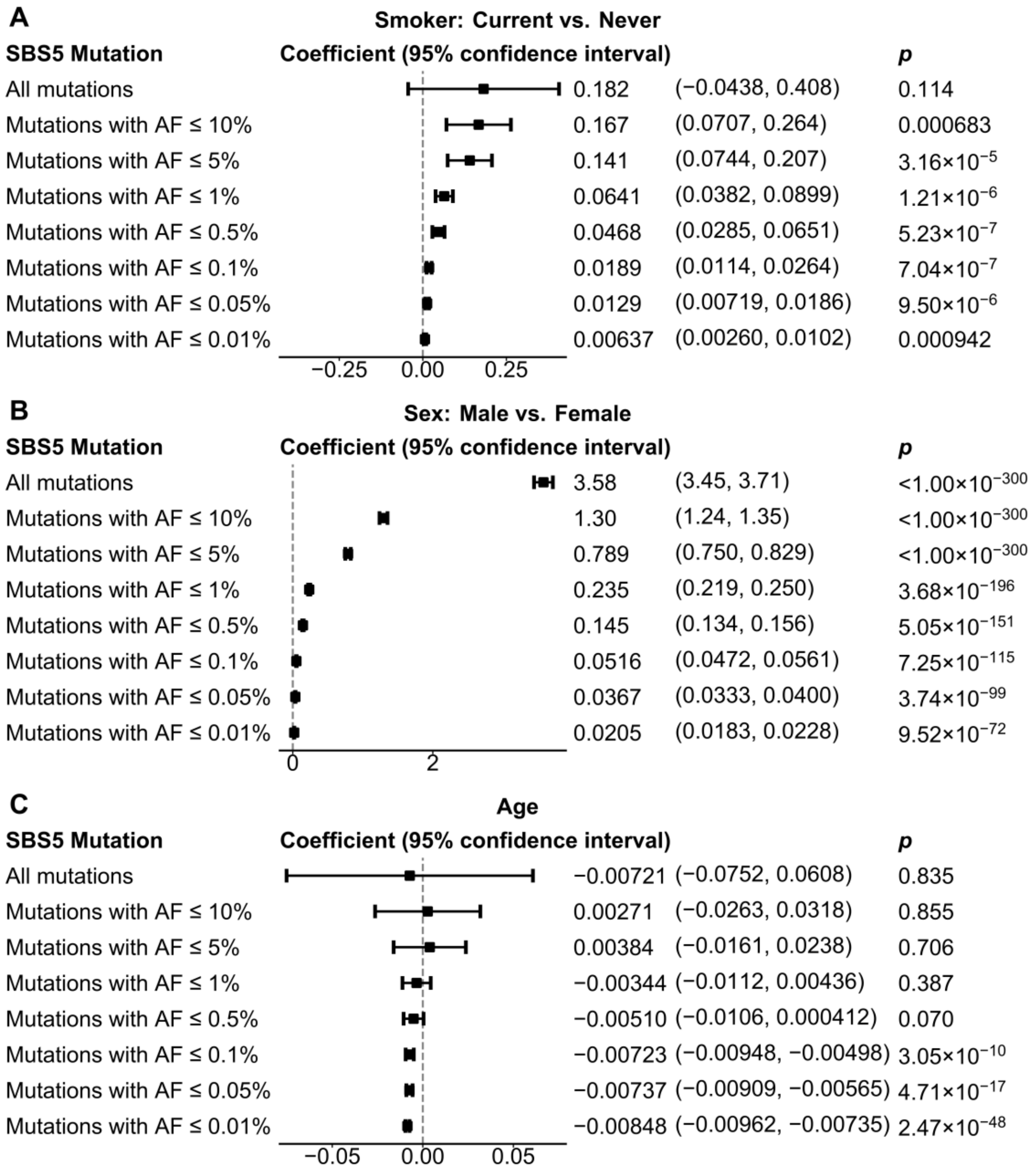

Supplementary Figure S6. Multivariable analysis of mutagenic factors influencing SBS5-related germline mutation burdens. This figure displays forest plots from multivariable linear regression models, examining the associations of SBS5-related germline mutation burden with (**A**) smoking status, (**B**) sex, and (**C**) age under different allele frequency (AF) thresholds. The square points represent the estimates of the regression coefficients, and the horizontal lines indicate the 95% confidence intervals (CIs). For smoking status, "never smoking" was used as the reference group, while "female" is the reference for the sex variable. The coefficients, 95% CIs, and *p*-values for each variable are listed to the right of each panel. Please refer to Table 1 for the detailed statistics of mutation burdens corresponding to each AF threshold. The number of individuals included in the multivariable linear regression analysis was 440,759.

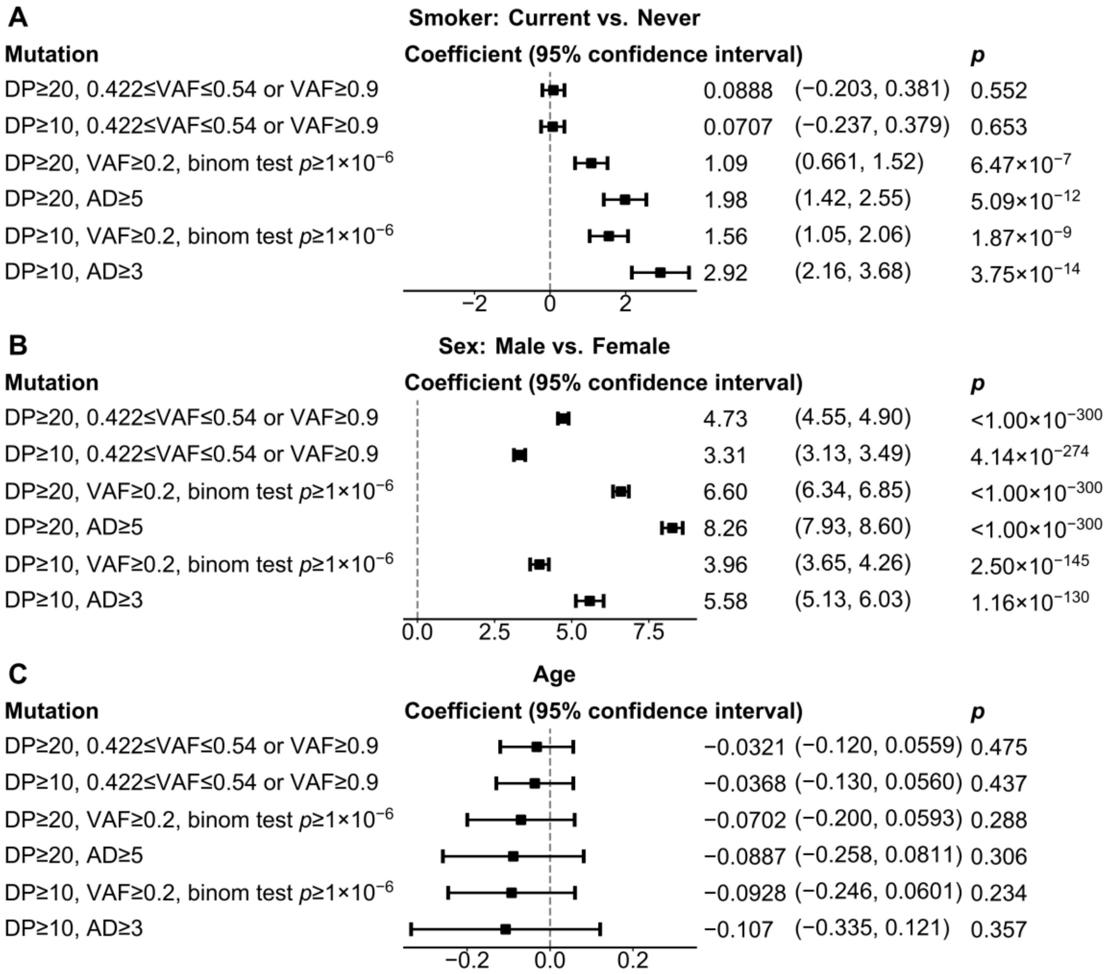

Supplementary Figure S7. Multivariable analysis of mutagenic factors influencing germline mutation burdens under different filtering strategies. This figure displays forest plots from multivariable linear regression models, examining the associations of germline mutation burden with (**A**) smoking status, (**B**) sex, and (**C**) age under different mutation filtering strategies. The square points represent the estimates of the regression coefficients, and the horizontal lines indicate the 95% confidence intervals (CIs). For smoking status, "never smoking" was used as the reference group, while "female" is the reference for the sex variable. The coefficients, 95% CIs, and *p*-values for each variable are listed to the right of each panel. The number of individuals included in the multivariable linear regression analysis was 440,759.

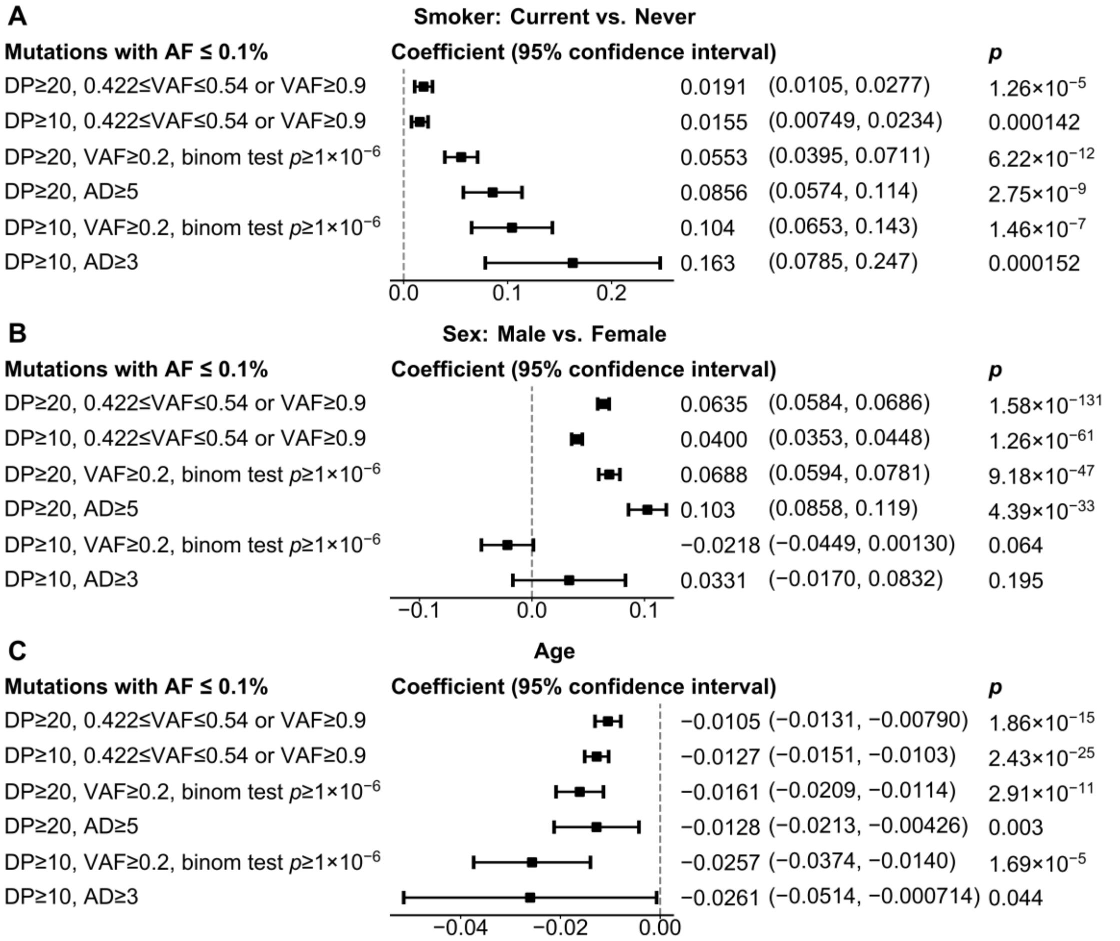

Supplementary Figure S8. Multivariable analysis of mutagenic factors influencing rare germline mutation burdens under different filtering strategies. This figure displays forest plots from multivariable linear regression models, examining the associations of rare (allele frequency ≤ 0.1%) germline mutation burden with (**A**) smoking status, (**B**) sex, and (**C**) age under different mutation filtering strategies. The square points represent the estimates of the regression coefficients, and the horizontal lines indicate the 95% confidence intervals (CIs). For smoking status, "never smoking" was used as the reference group, while "female" is the reference for the sex variable. The coefficients, 95% CIs, and *p*-values for each variable are listed to the right of each panel. The number of individuals included in the multivariable linear regression analysis was 440,759.
